# Genetics, Morphometrics and Health Characterization of Green Turtle Foraging Grounds in Mainland and Insular Chile

**DOI:** 10.3390/ani12121473

**Published:** 2022-06-07

**Authors:** Rocío Álvarez-Varas, Carol Medrano, Hugo A. Benítez, Felipe Guerrero, Fabiola León Miranda, Juliana A. Vianna, Camila González, David Véliz

**Affiliations:** 1 Center for Ecology and Sustainable Management of Oceanic Islands (ESMOI), Departamento de Biología Marina, Universidad Católica del Norte, Coquimbo 1780000, Chile; dveliz@uchile.cl; 2 Qarapara Tortugas Marinas Chile NGO, Santiago 7750000, Chile; camedrano@uc.cl (C.M.); fgb.guerrero@gmail.com (F.G.); 3 Laboratorio de Ecología y Morfometría Evolutiva, Centro de Investigación de Estudios Avanzados del Maule, Universidad Católica del Maule, Talca 3466706, Chile; hbenitez@ucm.cl; 4 Centro de Investigación en Recursos Naturales y Sustentabilidad (CIRENYS), Universidad Bernardo O’Higgins, Santiago 8370993, Chile; 5 Departamento de Ecosistemas y Medio Ambiente, Facultad de Agronomía e Ingeniería Forestal, Pontificia Universidad Católica de Chile, Santiago 8940000, Chile; lafabileon@gmail.com (F.L.M.); jvianna@uc.cl (J.A.V.); 6 Millennium Institute Center for Genomic Regulation (CRG), Santiago 7800003, Chile; 7 Centro de Estudios Avanzados en Zonas Áridas (CEAZA), Coquimbo 1780000, Chile; cam.gonzalezj@gmail.com; 8 Departamento de Ciencias Ecológicas, Facultad de Ciencias, Universidad de Chile, Santiago 7800003, Chile

**Keywords:** *Chelonia mydas*, regional connectivity, geometric morphometrics, Eastern Pacific, Polynesia, Easter Island, marine protected areas

## Abstract

**Simple Summary:**

Chilean waters constitute a foraging habitat for the endangered green turtle. Information about this species in the country has increased in recent years; nevertheless, little is known of its ecology and health status. Additionally, some populations have drastically decreased, probably due to human factors. Here, we studied the proportion of sex, age, morphological variation, genetic characteristics, origin, and health status of green turtles in mainland and insular Chile. We found that turtles from both regions are morphologically and genetically different. Individuals from the mainland territory are juveniles and probably originated from Galapagos. In contrast, the insular territory hosts juveniles and adults that probably originated from Galapagos and French Polynesia. We also found that turtles from both regions are facing numerous anthropic threats that must be controlled. We suggest the creation of protected areas for mainland foraging grounds, and strengthen the administrative plan of the insular region to ensure sea turtle population health.

**Abstract:**

Two divergent genetic lineages have been described for the endangered green turtle in the Pacific Ocean, occurring sympatrically in some foraging grounds. Chile has seven known green turtle foraging grounds, hosting mainly juveniles of different lineages. Unfortunately, anthropic factors have led to the decline or disappearance of most foraging aggregations. We investigated age-class/sex structure, morphological variation, genetic diversity and structure, and health status of turtles from two mainland (Bahia Salado and Playa Chinchorro) and one insular (Easter Island) Chilean foraging grounds. Bahia Salado is composed of juveniles, and with Playa Chinchorro, exclusively harbors individuals of the north-central/eastern Pacific lineage, with Galapagos as the major genetic contributor. Conversely, Easter Island hosts juveniles and adults from both the eastern Pacific and French Polynesia. Morphological variation was found between lineages and foraging grounds, suggesting an underlying genetic component but also an environmental influence. Turtles from Easter Island, unlike Bahia Salado, exhibited injuries/alterations probably related to anthropic threats. Our findings point to establishing legal protection for mainland Chile’s foraging grounds, and to ensure that the administrative plan for Easter Island’s marine protected area maintains ecosystem health, turtle population viability, and related cultural and touristic activities.

## 1. Introduction

Sea turtles are highly mobile species with a complex life history characterized by extensive migrations of adults from foraging grounds (FGs) to distant nesting areas (rookeries) and movements of juveniles among a variety of marine habitats [1]. Although these species spend most of their time in FGs, a higher proportion of ecological research has been focused on rookeries [2,3,4]. Only in the last few decades has there been an increase in the investigation of sea turtle FGs [3,4].

The green turtle (*Chelonia mydas*) is a cosmopolitan species listed as globally endangered in the IUCN Red List [5]. In the Pacific Ocean, its nesting rookeries are located in tropical and subtropical areas, but its FGs extend to temperate zones at high latitudes in both hemispheres [6,7]. Previous studies in the Pacific suggest the presence of two *C. mydas* divergent genetic lineages based on mitochondrial DNA that are reproductively isolated but sympatric in some FGs [8,9,10]. These lineages vary in their body shape (head, carapace, plastron, and flippers) and also in their coloration [8,9,10,11]. Currently they are known as the north-central/eastern Pacific lineage, or black morphotype; and the south-central/western Pacific lineage, or yellow morphotype [8,9,10,12].

Recent research using single-nucleotide polymorphisms (SNPs) suggested that genetic divergence between these morphotypes probably arose from strong male and female natal-homing behavior [12]. Furthermore, genes and enriched biological functions linked to thermoregulation, hypoxia, melanism, morphogenesis, osmoregulation, diet, and reproduction showed significant differences in the allelic frequencies, aligning with ecological differences reported between both morphotypes. These findings provide evidence for adaptation of this species to the Eastern Pacific region and support the independent evolutionary trajectory of the black and yellow morphotypes [12].

Six neritic-aggregation areas have been described for green turtles in mainland Chile, all of them located in the north of the country: Playa Chinchorro (18°28′ S, 70°18′ W), Bahía Chipana (21°19′ S, 70°03′ W), Bahía Mejillones del Sur (23°05 ′S, 70°28 ′W), Caleta Constitución (23°24′ S, 70°35′ W), Poza Histórica de Antofagasta (23°35′ S, 70°23′ W), and Bahía Salado (27°41′ S, 70°59′ W) [7]. For insular territory, there is evidence of this species’ aggregations in Easter Island (Rapa Nui, 27°09′ S, 109°26′ W) [13] and Juan Fernández Archipelago (Paulina Stowhas pers. comm, 33°38′ S, 78°50′ W). All of these areas include relatively sheltered locations with soft-bottom habitats dominated by macroalgae [14,15]. In particular, Bahia Salado is dominated by macroalgae and an endemic and endangered seagrass, *Zostera chilensis* [7,16].

Previous studies indicate that most individuals from *Chelonia mydas* Chilean aggregations are juveniles [7,15]. The northernmost FGs, where water temperatures are higher, also harbor adult turtles that include a higher proportion of females [17]. On the other hand, mainland Chilean FGs seem to exclusively host turtles corresponding to the black morphotype. Up to now, the yellow morphotype has only been reported on Easter Island [13].

Unfortunately, today most of the *C. mydas* Chilean aggregations seem to have suffered alterations in their population sizes. Some have disappeared due to several factors, including bycatch, marine pollution, and sea lion attacks [15,18,19,20,21]. Playa Chinchorro, recognized as the most important Chilean FG, has seen a drastic decrease in their abundance since 2017, when turtles started appearing headless and/or without limbs with no apparent cause [22]. Coastal aggregations from Bahia Chipana and the Antofagasta region (Bahía Mejillones del Sur, Caleta Constitución, and Poza Histórica de Antofagasta) have completely disappeared due to sea lion attacks and possibly bycatch [18,20]. In these areas today, only a few individuals can be sighted offshore [23]. Bahia Salado, a small bay described as the southernmost FG for the species, currently faces habitat degradation associated with intense boat traffic and uncontrolled macroalgae extraction. In Easter Island, a recently described FG for *C. mydas*, the population status is currently unknown despite reports of several anthropogenic threats (boat collision and marine pollution, among others) [13].

Ecological and health data are crucial for planning conservation strategies for the sea turtle species and their habitats in Chilean waters [7,24]. Here, we provide new information in terms of ecology and health for three *C. mydas* aggregations in Chile, including Easter Island, from which there are no data to date. Specifically, we report genetic diversity and population structure of these three aggregations (Playa Chinchorro, Bahia Salado, and Easter Island) using both mtDNA and single-nucleotide polymorphisms (SNPs). Likewise, we describe the age-class/sex structure, morphological variation, and health status of Bahia Salado and Easter Island’s turtles. This information should be taken into account to protect the few natural aggregations that still persist in Chile, considering the anthropogenic factors that threaten them.

## 2. Materials and Methods

### 2.1. Data Collection

**Figure 1:**
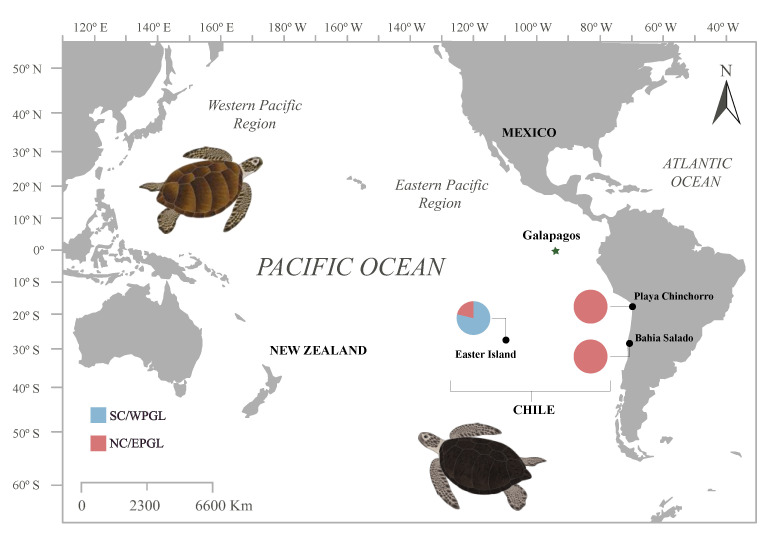
Map depicting the *Chelonia mydas* Pacific foraging grounds included in this study. Pie charts indicate the proportions of each genetic lineage within the foraging grounds. SC/WPGL, south-central/western Pacific genetic lineage; NC/EPGL, north-central/eastern Pacific genetic lineage.

*Chelonia mydas* individuals were captured from two FGs: Bahía Salado (27°41′ S, 70°59′ W), located in the north of mainland Chile (Atacama Region) and Hanga Roa, Easter Island (Rapa Nui; 27°09′ S, 109°26′ W) situated 3700 km offshore in the Valparaíso Region (Figure 1). Twenty turtles were captured in Bahia Salado between 2013 and 2021 using an entanglement net (50 × 1.8 m, mesh size of 35 cm stretched), which was checked constantly from the coastline by two apnea divers every 30 min. Twenty individuals were captured in Hanga Roa by hand by trained local divers during November 2018. In all cases, turtles remained out of the water no more than 40 min and prior to release were tagged on each front flipper using Inconel tags (Style 681, National Band and Tag Company, Newport, KY, USA [25]). Body measurements, weight, and skin samples were collected from each individual. Twenty-seven turtles from Playa Chinchorro (18°28′ S, 70°18′ W; Arica and Parinacota Region) were found stranded on the beach between 2017 and 2018. The cause of death was unknown, but most of them exhibited flippers and/or head muscle tearing. From these turtles, only skin samples were collected for genetic analyses given their advanced stage of decomposition.

Captures in Bahia Salado and Easter Island were authorized by the Chilean Sub-Secretariat of Fishing (SUBPESCA, by its Spanish abbreviation), through a Research Capture Permit (Exempt Resolution N°917, N°3712, N°3830, N°2021-089 for Bahia Salado and N°3755 for Easter Island). In addition, in Easter Island, captures were authorized by the local community though the “Consejo del Mar”. Samples from Playa Chinchorro turtles were collected through a special permit issued by Chilean Fisheries and Aquaculture Service (SERNAPESCA).

### 2.2. Traditional Morphometric Data

Morphometric data were collected for turtles from Bahia Salado and Easter Island. The following measurements were taken for each turtle: minimum curved carapace length (CCL min), curved carapace length notch to tip (CCL), curved carapace width (CCW), straight carapace length notch to tip (SCLn-t), straight carapace width (SCW), plastron length (PL), plastron width (PW), head length (HL), head width (HW), tail total length (TTL), and post-cloacal tail length (PTL) [2]. Curved and straight measurements were obtained using a metric tape and a calibrated forester’s caliper (0.1 cm, straight measurements), respectively. Body mass was obtained using a digital scale (±0.1 kg).

### 2.3. Life-Stage Determination

Given that black and yellow morphotypes reach different sizes in adults, the classification of an individual’s life stage (juvenile/adult) was based on their natal origin according to their mitochondrial haplotype (genetic lineage; see mtDNA analysis below [10]). Based on the mean CCL size of nesting females in Galapagos, black turtles (north-central/eastern morphotype) with CCL < 85 cm were classified as juveniles and CCL ≥ 85 cm as adults [25]. For yellow turtles (south-central/western morphotype), based on the CCL size of nesting females in French Polynesia, those individuals with CCL < 94 cm were classified as juveniles and CCL ≥ 94 cm as adults [26]. Turtles with TTL ≥ 25 cm were classified as males [27].

### 2.4. Body Condition Index

A body condition index (BCI = (body mass/SCLn-t^3^) × 10,000 [28]) was calculated to evaluate the relative “fatness” of turtles from Bahia Salado and Easter Island. This index was used as an indirect predictor of the nutritional status and/or health condition of animals [7,28]. BCI was calculated according to genetic lineage and FG.

### 2.5. Physical Examination

The attitude, activity level, eyelid reflex, threat response, respiratory activity, and hydration status of turtles from Bahia Salado and Easter Island were assessed by a veterinarian following the protocol described by [29,30]. A visual examination was performed from proximal to caudal portion of each individual. The eyes, ears, nasal cavity, and oral cavity were evaluated to detect signs of lesions or trauma (asymmetry, secretions, wounds, abnormal smell, presence of hooks or fishing line, presence of epibionts, etc.). In the carapace and plastron, changes in the firmness and flexibility were evaluated as well as injuries caused by boat propellers, ulceration, shark bites, deformities, fibropapillomatosis, and fractures. In addition, the presence of epibionts was registered, and they were carefully removed whenever possible. The mobility, muscle tone, and strength of flippers were evaluated. In addition, signs of inflammation, crackling, or skeletal deformities were registered. The tail and cloaca were examined in search of foreign materials, masses, protuberances, or prolapse.

### 2.6. Geometric Morphometrics

Geometric morphometric (GM) analyses included individuals from Bahia Salado and Easter Island. GM analyses were focused on variation in carapace shape and were performed using dorsal photographs with a reference scale. Thirty-six landmarks were digitized with TPS Dig v2.30 [31]. Landmarks were obtained between specific carapace scutes and at the borders of the marginal scutes [9,10]. In order to remove any nonshape elements, a Procrustes superimposition was applied to the landmark data [32]. To determine the influence of size on shape (allometry) in the dataset and to avoid any error influence by size (juveniles/adults), a multivariate regression was carried out [9,10]. Furthermore, a permutation test using 10,000 iterations was performed to assess the significance of the influence of the size on shape.

To visualize the variation in carapace shape, the average carapace shape was computed for each genetic lineage (based on mitochondrial haplotype; see mtDNA analysis below) and FG. Principal component analyses (PCA) were performed using the covariance matrices of shape variation and the average shape variation in genetic lineages and FG. To obtain a better graphical representation of the data and to discriminate groups based on carapace shape variation, a canonical variate analysis (CVA) was performed contrasting genetic lineages and FGs [9,10]. The results were reported as Mahalanobis distances and the respective *p*-values for these distances, after permutation tests (10,000 iterations).

Analyses were performed using MorphoJ v1.6 [33], and the package MOMOCS [34] and geomorph using the gmShiny desktop tool for R [35]. For these analyses, data were pooled according to genetic lineage (north-central/eastern Pacific, *n* = 24; south-central/western Pacific, *n* = 15) and FG (Bahia Salado, *n* = 20; Easter Island, *n* = 20).

### 2.7. Mitochondrial DNA Analysis

A skin sample (5 mm) of turtles from Playa Chinchorro (*n* = 27), Bahia Salado (*n* = 15) and Easter Island (*n* = 20) was collected using a sterile scalpel. Samples were stored in ethanol at room temperature and used for mtDNA and genomic (single-nucleotide polymorphisms-SNPs) analyses.

Mitochondrial DNA (mtDNA) control-region haplotypes were amplified in order to assign the putative genetic lineage (north-central/eastern Pacific and south-central/western Pacific, according to [10]), and to characterize diversity and genetic structure of each genetic lineage and FG. DNA was isolated using a modified protocol from that described by [36]. DNA quality and concentration were determined with a specific fluorimetry method using a Qubit fluorimeter (Life Technologies, Grand Island, NY, USA). The control region (D-loop; approx. 773 bp) was amplified using primers LCM15382 (5′ GCT TAA CCC TAA AGC ATT GG 3′) and H950g (5′ GTC TCG GAT TTA GGG GTT TG 3′) described by [37]. Polymerase chain reaction (PCR) protocol was carried out as described by [7]. The PCR product was visualized using electrophoresis in 1% agarose with red gel. The previously described procedures were performed at the Laboratorio de Biodiversidad Molecular, Departamento de Ecosistemas y Medio Ambiente, Pontificia Universidad Católica de Chile, Santiago, Chile. Final products were purified and sequenced bilaterally at Macrogen Inc., Seoul, Korea.

Raw sequences were edited and corrected manually using the SEQUENCHER v.5.4.6 (Gene Codes Corporation, Ann Arbor, MI, USA) and truncated to the standard length of 765 bp. Sequences were aligned using ClustalX v.2.1 [38] and haplotypes were identified after running a BLAST search implemented in the GenBank database (National Center for Biotechnology Information, Bethesda, MD, USA: NCBI Home page http://www.ncbi.nlm.nih.gov; accessed on 15 May 2020).

A median-joining network (MJN) was constructed in Network v10.2 [39] in order to visualize genetic diversity and possible geographic association among haplotypes. Genetic diversity was estimated by calculating the following summary statistics in ARLEQUIN v.3.5 [40]: number of polymorphic sites (S), haplotype number (h), haplotype diversity (Hd) and nucleotide diversity (π) for each genetic lineage and FG. Finally, by considering the same groups, pairwise FST were calculated to assess population structure using ARLEQUIN v.3.5. All these analyses were based on 61 samples, as one sample from Easter Island presented unsuccessful amplification.

### 2.8. Genomic Analysis

After DNA quality determination, 41 samples with high DNA quality (Playa Chinchorro, *n* = 16; Bahia Salado, *n* = 13; and Easter Island, *n* = 12) were sent for sequencing and genotyping using DArTseq TM genotyping technology to Diversity Arrays Technology in Canberra, Australia [41]. Library preparation, sequencing, quality control, and initial SNP calling were carried out as described by [12].

Loci were identified as SNP or reference alleles according to the occurrence frequency. Further filtering on FASTQ files was conducted using the dartR package implemented in R software [42]. Filtering included the following criteria: reproducibility (threshold = 1.00), minor allele frequencies (MAF) > 0.01, individual call rate (proportion with non-missing scores for each individual, removing those individuals below a specified threshold) with threshold > 0.90, locus call rate (proportion with non-missing scores for each locus, removing those loci below a specified threshold) with threshold > 0.85, and discarding monomorphic markers. Fragments containing more than one SNP were filtered using the gl.filter.secondaries command implemented in the dartR library.

A Euclidian distance-based principal coordinates analysis (PCoA) was carried out with the dartR package in order to observe the genetic relationships among individuals. In addition, pairwise FST between genetic lineages and FGs was calculated using the gl.fst.pop command of the dartR package. Significance was assessed by 1000 bootstrap replicates. PCoA and pairwise FST analysis included all individuals (*n* = 41) and loci (3003 SNPs) recovered in this study.

## 3. Results

### 3.1. Lineal Morphology, Life Stage, and Body Condition

All turtles captured in Bahia Salado (*n* = 20) were juveniles, with CCL varying between 42.4 and 83.1 cm (mean size of 62.12 ± 10.30 cm) and weighing between 11.8 and 76 kg (mean of 35.27 ± 17.34 kg; Table 1). The body condition index (BCI) ranged between 1.27 and 2.46 (mean of 1.74 ± 0.29). A total of 20 individuals were captured at Easter Island: 16 juveniles, 3 adult females, and 1 adult male. The CCL varied between 49.0 and 99.0 cm (mean size of 70.28 ± 13.11 cm) with weights between 15.3 and 138.0 kg (mean of 51.11 ± 31.47 kg; Table 1). The BCI of Easter Island turtles ranged between 1.62 and 2.37 (mean of 1.85 ± 0.19). Life stage, morphological, and BCI data for turtles from both FGs are shown in Table 1. When comparing genetic lineages (NC/EPGL, *n* = 24 and SC/WPGL, *n* = 15), we observe a mean CCL of 63.31 ± 10.29 and 69.71 ± 14.40, weight of 37.65 ± 16.2 and 51.56 ± 34.88, and BCI of 1.76 ± 0.27 and 1.85 ± 0.21, for NC/EPGL and SC/WPGL, respectively (Table 1).

### 3.2. Geometric Morphometrics

Given that the multivariate regression showed a 5% of allometry with a significant permutation value (*p*-value ≤ 0.01), a correction for allometry was performed and all the shape analyses (PCA and CVA) were carried out using the covariance matrix of the data corrected by size [9,10]. Therefore, in this study a distinction between juveniles and adults was not performed for geometric morphometric analyses.

**Figure 2:**
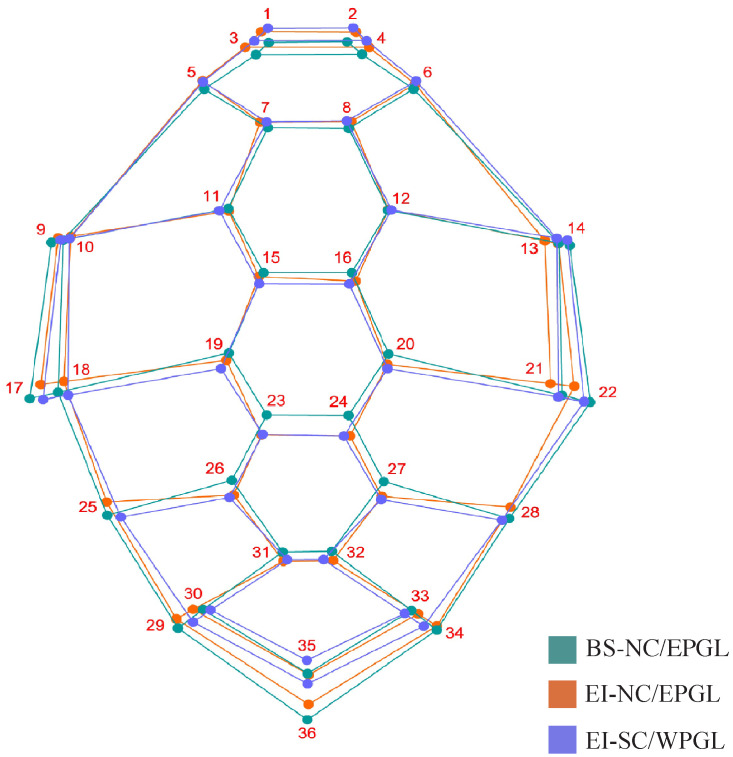
Average carapace shape between two *Chelonia mydas* foraging grounds (Bahia Salado and Easter Island) and their corresponding genetic lineages (NC/EPGL and SC/WPGL). Numbers represent landmarks. Colors represent foraging grounds and genetic lineages. Green: north-central/eastern Pacific genetic lineage turtles from Bahia Salado; orange: north-central/eastern Pacific genetic lineage turtles from Easter Island; and blue: south-central/western Pacific genetic lineage turtles from Easter Island.

The geometric shape of *C. mydas* carapace and its individual variation is shown in Figure 2. NC/EPGL turtles from Bahia Salado (Atacama) exhibited a triangular carapace with the elongation vector at the right of landmarks 13 and 21 and at the left of landmarks 10 and 18, and an anteroposterior narrowing of the second lateral scute (landmarks 10–13, 15, 16, and 18–21) in comparison with the other groups (Figure 2). Furthermore, they showed an expansion of the landmark 36 in the caudal carapace portion. In contrast, NC/EPGL turtles from Easter Island exhibited an elongation in the upper (proximal) portion of the carapace composed by landmarks 1–4 and a contraction of the lateral carapace landmarks (9, 10, 13, 14, 17, 18, 21, 22). The SC/WPGL turtles from Easter Island exhibited an oval carapace, with the upper portion even more elongated but with a marked contraction in the lower (caudal) carapace portion (landmark 35 and 36). They also exhibited central scutes slightly wider and lateral scutes narrower than the other groups (Figure 2).

**Figure 3:**
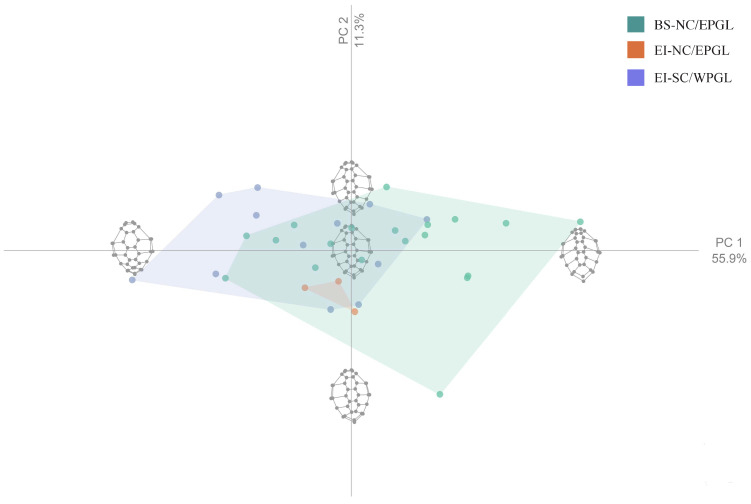
Principal component analysis (PCA) between two *Chelonia mydas* foraging grounds (Bahia Salado and Easter Island) and their corresponding genetic lineages (NC/EPGL and SC/WPGL). PCs represent each principal component. The wireframe of the carapace represents the shape of the maximum of each component. Colors represent foraging grounds and genetic lineages. Green: north-central/eastern Pacific genetic lineage turtles from Bahia Salado; orange: north-central/eastern Pacific genetic lineage turtles from Easter Island; and blue: south-central/western Pacific genetic lineage turtles from Easter Island.

The shape variation of the PCA accounted for 76.5% in the first three components (PC1: 56.04%, PC2: 11.3%, and PC3: 9.14%). In the scatterplot, although there is overlapping of some points, two large groups are observed corresponding to each genetic lineage (NC/EPGL and SC/WPGL; Figure 3). NC/EPGL turtles from Easter Island (*n* = 3) grouped together, composing a small group between both genetic-lineage-based large groups (Figure 3).

**Figure 4:**
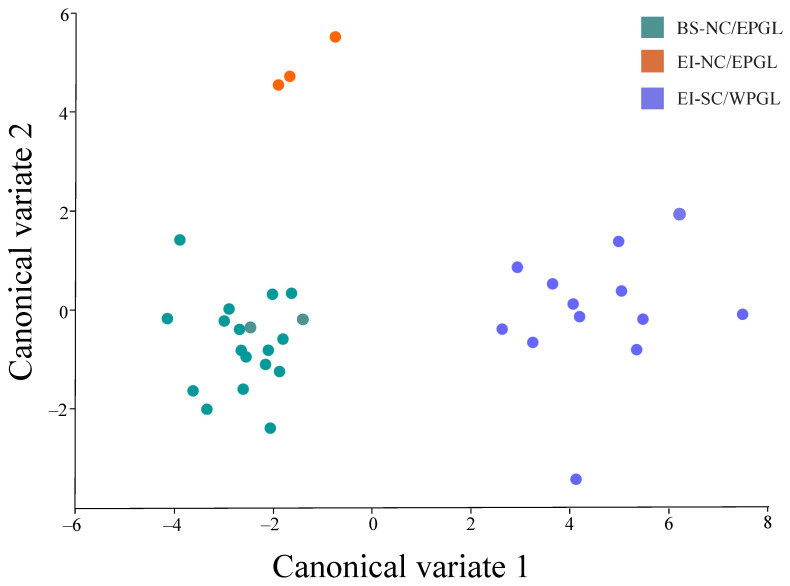
Canonical variate analysis (CVA) between two *Chelonia mydas* foraging grounds (Bahia Salado and Easter Island) and their corresponding genetic lineages (NC/EPGL and SC/WPGL). Colors represent foraging grounds and genetic lineages. Green: NC/EPGL from Bahia Salado; orange: NC/EPGL from Easter Island; and blue: SC/WPGL from Easter Island.

The first axis of the CVA (CV1) segregated both genetic lineages (NC/EPGL and SC/WPGL), and the CV2 separated populations within NC/EPGL (Bahia Salado and Easter Island; Figure 4). Mahalanobis distances among groups were significant for all three combinations: NC/EPGL from Bahia Salado vs. NC/EPGL from Easter Island (*p*-value = 0.04), NC/EPGL from Bahia Salado vs. SC/WPGL from Easter Island (*p*-value = 0.05), and NC/EPGL from Easter Island vs. SC/WPGL from Easter Island (*p*-value = 0.04).

### 3.3. Mitochondrial DNA Analysis

A total of 61 individuals were sequenced for the control region (CR, mtDNA), representing two FGs in mainland Chile (Playa Chinchorro, *n* = 27 and Bahia Salado, *n* = 15) and one in insular territory (Easter Island, *n* = 19). We recovered nine haplotypes for Playa Chinchorro, six for Bahia Salado, and seven for Easter Island (Table 2). All haplotypes from mainland Chile and four from Easter Island (CmP4.1, CmP4.14, CmP4.4, and CmP4.6) were classified as part of the north-central/eastern Pacific genetic lineage (NC/EPGL; Table S1) [10]. The three remaining haplotypes from Easter Island (CmP97.1, CmP109.1, and CmP207.1) were classified as part of the south-central/western Pacific genetic lineage (SC/WPGL; Table S1) [10].

In Playa Chinchorro, three endemic haplotypes from Galapagos, Ecuador (CmP4.9, CmP15.1, and CmP17.1) and one endemic haplotype from Michoacan, Mexico (CmP5.1) were found (Table S1) [10,43,44]. In previous studies, haplotypes CmP4.4, CmP4.6, and CmP4.7 and haplotype CmP4.1 have been reported with the highest frequency in Galapagos and Michoacan, respectively [43,44]. One haplotype (CmP93.2) was classified as “orphan” as it has not been reported at any Pacific rookery to date (Table S1) [10,44]. For Bahia Salado, we found one haplotype endemic to Galapagos (CmP15.1) and one endemic to Michoacan (CmP5.1; Table S1). Furthermore, three haplotypes have been reported with the highest frequency for Galapagos (CmP4.4, CmP4.6 and CmP4.7) and one haplotype with the highest frequency for Michoacan (CmP4.1) [43,44]. No orphan haplotypes were reported for this FG. Finally in Easter Island, two haplotypes were endemic to Galapagos (CmP4.4 and CmP4.6) and one to Michoacan (CmP4.1; Table S1). Eleven individuals carried the haplotype CmP97.1, which was recently described in nesting sites from French Polynesia [45]. In addition, three haplotypes were classified as orphans: CmP4.14 reported in New Zealand FG [10] and CmP109.1 and CmP207.1 found in Palmyra Atoll FG (Table S1) [46].

The number of polymorphic sites (S) varied between 17 and 47, being highest in Easter Island and lowest in Bahia Salado (Table 2). The haplotype diversity (Hd) varied between 0.66082 and 0.86040, showing the highest values in Playa Chinchorro and the lowest ones in Easter Island (Table 2). The nucleotide diversity (π) oscillated between 0.00202 and 0.01876, being higher in Easter Island and lower in Bahia Salado (Table 2). Regarding the genetic lineages, NC/EPGL (*n* = 46, Table 2) exhibited a higher number of haplotypes and haplotype diversity (h = 10, Hd = 0.83865) and a lower nucleotide diversity (π = 0.00223) than SC/WPGL (*n* = 15, h = 3, Hd = 0.44720, and π = 0.00617; Table 2).

**Figure 5:**
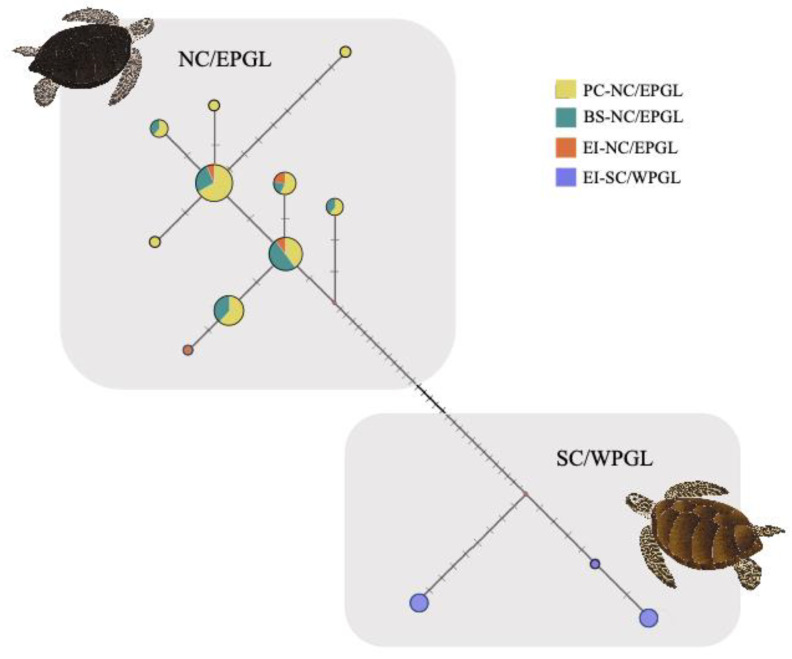
Median-joining network (MJN) according to three *Chelonia mydas* foraging grounds. The size of the circles is approximately proportional to haplotype frequency in the dataset. Yellow: north-central/eastern Pacific genetic lineage turtles from Playa Chinchorro; green: north-central/eastern Pacific genetic lineage turtles from Bahia Salado; orange: north-central/eastern Pacific genetic lineage turtles from Easter Island; and blue: south-central/western Pacific genetic lineage turtles from Easter Island.

The median-joining network exhibited two divergent haplogroups well-separated by thirty-one mutation steps (Figure 5). One haplogroup was composed of haplotypes shared among the three FGs (Playa Chichorro, Bahia Salado, and Easter Island, consistent with NC/EPGL: haplotypes 1–10, Figure 5). The other haplogroup comprised haplotypes exclusively from Easter Island, which were congruent with those classified as SC/WPGL (haplotypes 11–13; Figure 5). The most frequent haplotypes for the NC/EPGL were CmP4.6 and CmP4.1 (GenBank accession number KC306647.1 and KC306666.1, respectively), and for SC/WPGL was Cm97.1 (GenBank accession number FJ917198).

Pairwise genetic differences (F_ST_) based mtDNA only showed significant values (*p*-value ≤ 0.01) between genetic lineages (F_ST_ = 0.32181 for Playa Chinchorro vs. Easter Island-SC/WPGL; F_ST_ = 0.36667 for Bahia Salado vs. Easter Island-SC/WPGL and F_ST_ = 0.37696 for Easter Island-NC/EPGL vs. Easter Island-SC/EWPGL).

### 3.4. SNPs Analysis

**Figure 6:**
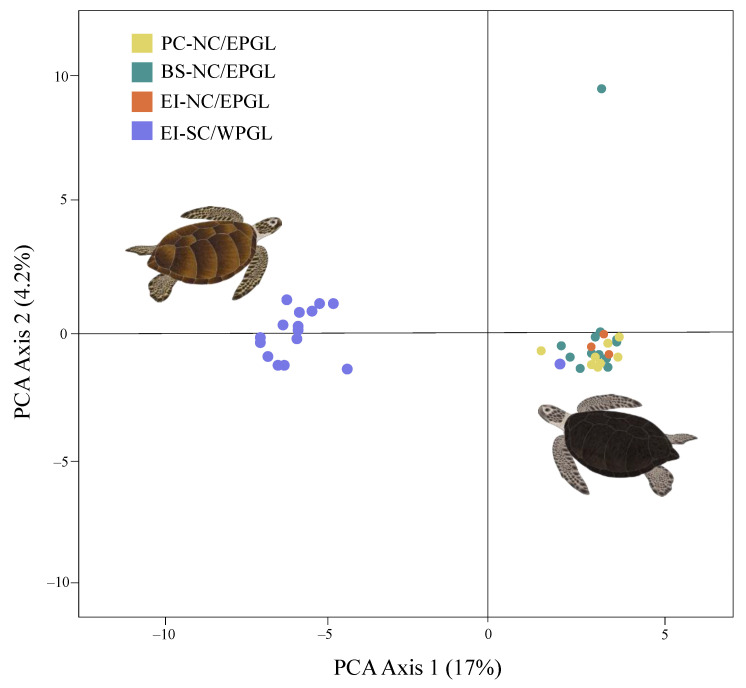
Principal coordinate analysis (PCoA) based on 3003 loci recovered from *Chelonia mydas* including individuals from three Chilean foraging grounds. Yellow: north-central/eastern Pacific genetic lineage turtles from Playa Chinchorro; green: north-central/eastern Pacific genetic lineage turtles from Bahia Salado; orange: north-central/eastern Pacific genetic lineage turtles from Easter Island; and blue: south-central/western Pacific genetic lineage turtles from Easter Island.

The PCoA exhibited a clear segregation of the genetic lineages in the first principal component (PC1 accounted for 17% of the total genetic variance) regardless of the FGs (Figure 6). Both groups exhibited slight data dispersion, except for one individual from Bahia Salado with lineage NC/EPGL. PC2 did not show clear difference among individuals (Figure 6).

Pairwise genetic differences (F_ST_) only showed significant values (*p*-value ≤ 0.01) between genetic lineages (F_ST_ = 0.141 for Playa Chinchorro vs. Easter Island-SC/WPGL; F_ST_ = 0.144 for Bahia Salado vs. Easter Island-SC/WPGL and F_ST_ = 0.141 for Easter Island-NC/EPGL vs. Easter Island-SC/WPGL).

## 4. Discussion

We investigated the ecology and health status of *Chelonia mydas* from three Chilean FGs. Our results showed that juveniles are more frequent in Chile, and insular waters host individuals from two divergent Pacific lineages (south-central/western Pacific-SC/WPGL and north-central/easter Pacific-NC/EPGL). Conversely, mainland FGs only seem to harbor turtles from the lineage NC/EPGL, also called “black morphotype”. These findings point to diverse natal origins, which together with anthropogenic threats reported in all these FGs highlight the relevance of protection.

### 4.1. Size Structure, Divergent Lineages, and Probable Genetic Contributors for Chilean Foraging Grounds

Our results show that turtles from Bahia Salado (mainland Chile) were all juveniles, whereas in Easter Island both juveniles and adults were reported. However, all adults of the island corresponded to SC/WPGL (yellow morphotype). Numerous studies have reported long-distance migration of adult *C. mydas* between their rookeries and foraging grounds in the South Pacific, proposing that factors such as food availability, ocean currents, the Earth’s geomagnetic field, and the passive drift experienced by hatchlings (during the “lost years”) could influence this behavior [47,48,49]. This is in line with our findings, since the most frequent SC/WPGL haplotype in Easter Island (CmP97.1) was recently described in French Polynesia [45], located at around 3400 km from Easter Island. In the same way, SCWPGL turtles that carried orphan haplotypes (CmP109.1 and CmP207.1), probably come from other Polynesian islands that have still not been genetically sampled (e.g., Pitcairn Islands; see [45,50,51]). This hypothesis arises due to such orphan haplotypes grouping in the same clade or a sister clade (see [10,46]).

Seminoff et al. [52] suggested that waters ≤ 25 °C may represent the thermal threshold, below which migrating adult females actively avoid surface waters in the Pacific Ocean. Unlike adults, juvenile green turtles can reach FGs with cooler water temperatures [7,53]. Bahia Salado reaches a maximum temperature of 20.5 °C in summer, while Easter Island can reach 25 °C during the same season [54,55]. Thus, the elevated surface water temperatures in Easter Island could be facilitating the permanence of SC/WPGL adult turtles in this isolated place, and lower temperatures in Bahia Salado could be tolerated by NC/EPGL juveniles. Likewise, these results are concordant with the characteristics of each morphotype. A recent investigation based on genomic data suggests that black turtles withstand lower water temperatures compared to yellow turtles [12] which is also supported by the widest distribution range of black turtles in Pacific temperate waters [6,7].

For the case of NC/EPGL turtles found in Easter Island and both mainland FGs (Playa Chinchorro and Bahia Salado), the most frequent haplotypes corresponded to those reported as endemic or most common in rookeries from the Galapagos Archipelago (Table S1) [43,44]. Likewise, endemic (or common) haplotypes from the Michoacan rookery (Mexico) were found to a lesser extent (Table S1) [43]. Moreover, haplotype CmP4.6 has been reported in Costa Rica rookeries and haplotype CmP4.1 in Costa Rica and Revillagigedo Archipelago, Mexico, but in lower frequency [43,44].

Although the small sample used here hindered performing an appropriate analysis to assess the most probable natal origin of turtles from the studied locations (mixed stock analysis-MSA [1]), the results reported above could suggest Galapagos as the main source of individuals for Chilean FGs. These results are consistent with those reported by [14,56], who suggested Galapagos as the major contributor for Arica and Antofagasta green turtles in northern Chile. Our study also extends the limit of Galapagos genetic contribution to Easter Island in the South Pacific.

Finally, both NC/EPGL orphan haplotypes have been reported in New Zealand FGs (CmP4.14 and CmP93.2) supporting transpacific migrations performed by *C. mydas*, as reported by several authors [8,10,43,44,46,50].

### 4.2. Genetic Diversity, Population Structure, and the Extension of the Pacific Management Units

Median-joining network and F_ST_ results based on mtDNA exhibited genetic differences between lineages but not between FGs (Figure 5). One haplogroup was composed by haplotypes shared among the three studied locations (Playa Chichorro, Bahia Salado, and Easter Island, consistent with NC/EPGL) and the other one was comprised by haplotypes exclusively from Easter Island (congruent with SC/WPGL haplotypes). Similar results were obtained using SNPs where the PCoA and pairwise F_ST_ only showed differences between genetic lineages (Figure 6). Some authors have reported genetic differentiation among green turtle FGs, suggesting the distribution of haplotypes among FGs is nonrandom and may be determined by ocean currents, among other factors [57,58]. It is probable that the lack of genetic differentiation between FGs found here is due to the shared natal origin between the turtles.

When examining genetic diversity (Hd), our mtDNA results show higher values for Playa Chinchorro, followed by Bahia Salado and then Easter Island (Table 2; Figure 5). However, these results may be biased by the presence of one or two lineages in each FG. If we evaluate haplotype diversity between lineages, we observe that turtles from NC/EPGL have almost twice the diversity of SC/WPGL (Table 2; Figure 5). Although these findings point to a greater need for protection of the island (the only FG that hosts SC/WPGL turtles), they should be interpreted cautiously, as two of the three FGs studied here exclusively host NC/EPGL turtles.

As previously mentioned, Galapagos seems to be the major source of individuals for Chilean FGs including NC/EPGL turtles from Easter Island. Wallace et al. [59] delimited seven Regional Management Units (RMUs) for *C. mydas* in the Pacific Ocean based on biogeography data (nesting sites, population abundances and trends, population genetics, and satellite telemetry), among them the East Pacific RMU. Likewise, Dutton et al. [43] reported four management units (MUs) for the Eastern Pacific region: Revillagigedo (Mexico), Michoacán (Mexico), Costa Rica, and Galapagos (Ecuador) [43,44]. Like other recent genetic investigations [14,56], this study suggests that mainland Chilean FGs are part of the East Pacific RMU, and specifically the Galapagos MUs.

Easter Island represents a more complex scenario, as it harbors turtles from potentially different natal origins. As noted, most SC/WPGL turtles originate from French Polynesia [45]. Dutton et al. [50] classified French Polynesia as a distinctive MU in the South Pacific region. Nevertheless, the haplotype CmP97.1 has only been described in this place, but it groups into a clade of the West Indian Ocean and Southwest Pacific Ocean [45], which points to the need of further research to understand its origin and distribution. These findings together with the presence of two orphan haplotypes in Easter Island that group in the same (or sister, CmP109.1 and CmP207.1) clade highlights the importance to increase the genetic sample efforts in this part of the South Pacific to better understand connectivity and establish regional conservation strategies for this endangered species.

### 4.3. Genetic and Environmental Influence on Chelonia mydas Carapace Shape

Geometric morphometric analyses, including PCA, CVA, and Mahalanobis distances, showed a clear segregation on the carapace shape between genetic lineages (Figure 3 and Figure 4). This pattern has been previously reported in this species using a large dataset of turtles from Pacific and Atlantic FGs, and suggests a strong genetic influence on body shape [9,10]. NC/EPGL turtles exhibited a triangular carapace with a longer caudal scute, while SC/WPGL turtles showed an oval carapace (Figure 2). These findings are consistent with previous reports that highlight the carapace shape as a key characteristic that is visually differentiated between both morphotypes [8,60]. Further research is needed to understand if these traits have a relationship with each morphotype fitness.

Within NC/EPGL turtles, we also found shape differences that point to variation beyond genetics. Turtles from Easter Island had an elongation in the proximal portion of the carapace and a contraction of the lateral landmarks in comparison with turtles from Bahia Salado (Figure 2). Although the PCA showed an overlap of some points, NC/EPGL turtles from Easter Island grouped all together composing a small group (Figure 3). Likewise, the CVA clearly segregated NC/EPGL turtles from Easter Island and Bahia Salado (Figure 4) and the Mahalanobis distances were significantly different between these groups. Such results suggest that ecological or environmental conditions in these sites could also be influencing carapace shape in this species, specially by considering that turtles from both FGs carry haplotypes in common (Figure 5).

### 4.4. Anthropic and Natural Threats to Turtles in Chilean Foraging Grounds

The physical examinations performed on turtles from Bahia Salado and Easter Island suggest that the first aggregation is composed of visually healthy individuals with good body condition and low epibiont load. Most of them exhibited lesions and scars associated with rock abrasion, which is highly probable since the area dominated by seagrass and algae where turtles feed is shallow (0.5–1.5 m) [7]. Only a couple of turtles exhibited lesions, probably caused by fungus, and one individual presented cloacal prolapse, which can be caused by several factors (e.g., trauma, obesity, foreign bodies, among others) [61]. Although our physical examination revealed that most threats are not of anthropic nature, previous research had reported elevated pollutant levels in blood of Bahia Salado turtles, probably related with human activities such as boat traffic and aquaculture [7,55]. Thus, the integration of different clinical tools is crucial to investigate the health status of natural sea turtle aggregations and visual examinations should be cautiously interpreted.

On the other hand, turtles from Easter Island showed variable body condition with some turtles apparently dehydrated and emaciated, showing signs of fishing gear interaction, and lesions congruent with bacterial, fungal and/or viral infections associated with marine pollution [62,63,64]. A recent study found extensive fields of filamentous cyanobacteria-like mats covering sandy substrates and dead reefs off Hanga Roa, probably derived from an ongoing eutrophication process as a consequence of over-tourism, absence of wastewater collection and treatment system, and the unlined landfill [65]. It is probable this situation has generated health issues in resident turtles, which require further research specifically linking environmental pollution with turtle blood/tissue alterations (heavy metals, organic pollutants, hormonal disorders, among others).

Half of the individuals from Easter Island exhibited carapace wounds caused by boat propellers. Previous studies showed that boat strikes pose a significant threat to sea turtles, mainly in touristic places. Denkinger et al. [66] through the underwater observation of live green turtles in San Cristóbal Island (Galapagos, Ecuador) reported that 19.4% of turtles (*n* = 124) exhibited carapace lesions caused by boat propellers. Likewise, Singel et al. [67] through stranding reports informed that 40% of sea turtles washed ashore had died from boat strikes in Florida, USA. Our findings highlight the relevance of establishing local actions aiming to decrease the impact caused by high artisanal-boat traffic in sea turtle feeding and resting areas in Easter Island. Reduction of boat speed limits in specific areas of the bay and requiring the use of propeller protectors are some actions recommended to decrease the number of fatal encounters between boats and turtles in the island [68].

Easter Island turtles exhibited a body condition index (BIC) of 1.85, while those from Bahia Salado had a lower BIC (1.74). Easter Island values are also higher when compared to other green turtle Pacific aggregations (see [7,17]). Hand-feeding turtles by fishermen and tourists seems to be a common practice, especially in touristic places [53,69,70]. Previous research indicates that this practice could lead to alterations in behavior and growth rates [69] and also predispose turtles to obesity, malnutrition, liver/renal disease, diabetes mellitus, gout, and cardiovascular issues [70]. Furthermore, turtles habituated to view humans as a food supplier increase the risk of injuries (such as boat strikes) and eventually the risk of capture in regions where open harvests for these species still exist [71]. Further evidence-based education of the local community and visitors is needed to avoid this detrimental practice with turtles as well as a long-term monitoring program to determine the health effects on this green turtle aggregation associated to this non-natural feeding.

### 4.5. The Relevance of Protecting Green Turtle Chilean FGs

In Bahia Salado, green turtles may remain for a long time (e.g., recaptures during nine consecutive years) and young (new) individuals are recruited every year [55], demonstrating its relevance as an FG for the species in the Eastern Pacific. Unfortunately, since 2008, several infrastructure projects have been proposed in the bay, which would threaten the local economy and coastal marine biodiversity. Among them, a multipurpose port to transport minerals, fertilizers, and heavy metals, among other products [72] and an electrical megaproject which comprises a terminal, gas duct, and a power plant that would be located in the middle of the bay [73]. Since 2013, Qarapara NGO has monitored the Bahia Salado ecosystem and has developed numerous efforts to protect this area; however, to date such efforts have been unsuccessful.

Until a few years ago, Playa Chinchorro represented the most important green turtle aggregation in Chile due to its high abundance [17]. However, there have not been turtle sightings in the area since 2018 (Alfredo Álvarez pers. comm). In 2011, a regional work group composed of local authorities, universities, and NGOs proposed the establishment of a Marine Reserve covering fifty-six hectares to ensure the protection of green turtles and its habitat in Arica [74]. Nevertheless, to date the legal resolution has been not adopted and the area remains unprotected.

In Easter Island, the Rapa Nui Multiple Uses Coastal Marine Protected Area (AMCP-MU), declared in 2018, covers 57.9 million hectares and is comanaged by Rapanui people and the Chilean Government [75]. The AMCP-MU administrative plan is the instrument that allows the management of the area, including strategies and actions, to be developed in terms of research, inspection, surveillance, outreach, management, and monitoring. This plan has recently been completed and will be implemented in the following years [75,76].

Our results demonstrate that the Chilean FGs are crucial to maintain the viability of regional green turtle populations. However, several anthropogenic factors currently threat these aggregations that in most cases are entirely unregulated and therefore unprotected. The worrying situation of Playa Chinchorro highlights the need to strengthen protections for the natural FGs that still remain in mainland and insular Chile. The health condition of Bahia Salado turtles, its high genetic diversity, and connectivity with the most important rookery in the region, highlights the importance of continued monitoring of this ecosystem and demand its legal protection in the short term in order to avoid the implementation of potentially detrimental industrial projects. Likewise, the poor health status of Easter Island turtles, the negative impacts of human activity, low genetic diversity, and high regional connectivity require the establishment of local management and conservation strategies to ensure the health of this aggregation in the long-term. Such strategies should be incorporated in the AMCP-MU administrative plan.

## Figures and Tables

**Table 1:** Morphometrics, weight, and BCI data (mean ± SD) of *Chelonia mydas* individuals by foraging ground and genetic lineage.

Variable	Foraging Ground		Genetic Lineage	
	Bahia Salado	Easter Island	NC/EPGL	SC/WPGL
CCL min	61.89 ± 10.30 (42.4–82.5)	69.91 ± 13.09 (49.0–99.0)	63.35 ± 10.26 (42.4–82.5)	69.71 ± 14.40 (49.0–99.0)
CCL	62.12 ± 10.30 (42.4–83.1)	70.63 ± 13.73 (49.0–99.0)	63.31 ± 10.29 (42.4–83.1)	70.51 ± 15.12 (49.0–99.0)
SCLn-t	57.33 ± 9.19 (38.7–76.3)	63.70 ± 12.36 (45.0–91.5)	58.38 ± 9.03 (38.7–76.3)	63.71 ± 13.58 (45.0–91.5)
CCW	59.77 ± 9.81 (39.7–79.1)	66.22 ± 13.30 (45.8–92.0)	60.90 ± 9.81 (39.7–79.1)	66.13 ± 14.44 (45.8–92.0)
SCW	47.18 ± 6.86 (32.5–62.4)	52.41 ± 9.58 (38.0–72.5)	47.93 ± 6.74 (32.5–62.4)	52.59 ± 10.52 (38.0–72.5)
HL	14.21 ± 2.05 (10.5–18.7)	15.60 ± 1.99 (12.6–19.9)	14.76 ± 2.36 (10.5–19.9)	15.09 ± 1.71 (12.6–18.6)
HW	9.22 ± 1.47 (6.9–12.0)	10.06 ± 1.51 (7.6–12.8)	9.57 ± 1.54 (6.9–12.6)	9.87 ± 1.52 (7.6–12.8)
TTL	10.99 ± 3.32 (6.5–20.8)	13.86 ± 4.49 (8.1–27.5)	11.76 ± 3.71 (6.5–20.8)	13.32 ± 4.74 (8.1–27.5)
PTL	3.54 ± 1.06 (1.8–6.4)	4.64 ± 1.38 (2.7–7.9)	3.82 ± 1.31 (1.8–7.5)	4.45 ± 1.31 (2.7–7.9)
PL	48.35 ± 7.34 (34.3–60.9)	50.28 ± 6.33 (39.6–60.8)	49.14 ± 7.15 (34.3–60.9)	49.42 ± 6.60 (39.6–60.8)
PW	48.29 ± 6.68 (36.0–57.0)	48.98 ± 6.24 (37.4–59.6)	48.33 ± 5.94 (36.0–57.2)	48.34 ± 6.60 (37.4–59.6)
Weight (Kg)	35.27 ± 17.34 (11.8–76.0)	51.11 ± 31.47 (15.3–138.0)	37.65 ± 16.2 (11.8–76.0)	51.56 ± 34.88 (15.3–138.0)
BCI	1.74 ± 0.29 (1.27–2.46)	1.85 ± 0.19 (1.62–2.37)	1.76 ± 0.27 (1.27–2.46)	1.85 ± 0.21 (1.63–2.37)
*n*	20	20	24	15

**Table 2:** Genetic indexes for *Chelonia mydas* according to foraging ground and genetic lineage.

Group		n	h	S	Hd	π
Foraging ground	Playa Chinchorro	27	9	17	0.86040	0.00238
	Bahia Salado	15	6	7	0.81905	0.00202
	Easter Island	19	7	47	0.66082	0.01876
Genetic lineage	NC/EPGL	46	10	18	0.83865	0.00223
	SC/WPGL	15	3	13	0.44720	0.00617
Total		61	13	60	0.87705	0.01762

## Data Availability

The data presented in this study are available on request from the corresponding author.

## Supplementary Materials

The following supporting information can be downloaded at: https://www.mdpi.com/article/10.3390/ani12121473/s1, Table S1: Biological and genetic data of *Chelonia mydas* from Chilean foraging grounds included in this study.
