# Supplementary material for: Genetics, Morphometrics and Health Characterization of Green Turtle Foraging Grounds in Mainland and Insular Chile
Source: Animals (Basel). 2022 Jun 7;12(12):1473. doi: 10.3390/ani12121473 (PMC9219523; doi:10.3390/ani12121473)
Supplement: Supplementary file 1 [file animals-12-01473-s001.zip › animals-1718152-supplementary.pdf]

Table S1. Biological and genetic data of *Chelonia mydas* from Chilean foraging grounds included in this study.

Location	ID	CCL	Life stage	Haplotype	Putative origin	Putative rookeries	Higher haplotype frequency	References
Bahia Salado	801AT	70.0	Juvenile	**CmP4.7**	Eastern Pacific	Galapagos, MNP (Ecuador)	Galapagos	[43, 44]
Bahia Salado	803AT	83.1	Juvenile	**CmP15.1**	Eastern Pacific	Galapagos (Ecuador)	Endemic Galapagos	[43, 44]
Bahia Salado	805AT	66.7	Juvenile	CmP4.7	Eastern Pacific			
Bahia Salado	807AT	19.2	Juvenile	**CmP4.1**	Eastern Pacific	Galapagos (Ecuador), MNP (Ecuador), Costa Rica, Michoacan (Mexico), Revillagigedo	Michoacan	[43, 44]
Bahia Salado	809AT	65.2	-	**CmP4.6**	Eastern Pacific	Galapagos (Ecuador), MNP (Ecuador), Costa Rica, Michoacan (Mexico)	Galapagos	[43, 44]
Bahia Salado	811AT	75.5	Juvenile	CmP4.6	Eastern Pacific			
Bahia Salado	813AT	71.0	Juvenile	CmP4.7	Eastern Pacific			
Bahia Salado	818AT	61.7	Juvenile	**CmP5.1**	Eastern Pacific	Michoacan	Endemic Michoacan	[43, 44]
Bahia Salado	820AT	50.3	Juvenile	CmP4.1	Eastern Pacific			
Bahia Salado	823AT	54.2	Juvenile	CmP4.1	Eastern Pacific			
Bahia Salado	826AT	71.8	Juvenile	CmP4.1	Eastern Pacific			
Bahia Salado	828AT	67.0	Juvenile	CmP4.6	Eastern Pacific			
Bahia Salado	831AT	54.7	Juvenile	**CmP4.4**	Eastern Pacific	Galapagos, MNP (Ecuador)	Galapagos	[43, 44]
Bahia Salado	837AT	55.3	Juvenile	CmP4.1	Eastern Pacific			
Bahia Salado	851AT	52.5	Juvenile	CmP4.6	Eastern Pacific			
Playa Chinchorro	3018ARI	-	-	**CmP4.1**	Eastern Pacific	Galapagos (Ecuador), MNP (Ecuador), Costa Rica, Michoacan (Mexico), Revillagigedo	Michoacan	[43, 44]
Playa Chinchorro	3118ARI	-	-	**CmP5.1**	Eastern Pacific	Michoacan	Endemic Michoacan	[43, 44]
Playa Chinchorro	3618ARI	-	-	**CmP4.6**	Eastern Pacific	Galapagos (Ecuador), MNP (Ecuador), Costa Rica, Michoacan (Mexico)	Galapagos	[43, 44]
Playa Chinchorro	3718ARI	-	-	CmP4.6	Eastern Pacific			
Playa Chinchorro	4118ARI	-	-	CmP4.1	Eastern Pacific			
Playa Chinchorro	4318ARI	-	-	CmP4.6	Eastern Pacific			
Playa Chinchorro	4418ARI	-	-	**CmP93.2**	Eastern Pacific	-	Orphan	[9, 44]
Playa Chinchorro	4718ARI	-	-	CmP4.6	Eastern Pacific			
Playa Chinchorro	4918ARI	-	-	**CmP17.1**	Eastern Pacific	Galapagos (Ecuador)	Endemic Galapagos	[43, 44]
Playa Chinchorro	6018ARI	-	-	**CmP15.1**	Eastern Pacific	Galapagos (Ecuador)	Endemic Galapagos	[43, 44]
Playa Chinchorro	6118ARI	-	-	**CmP4.7**	Eastern Pacific	Galapagos, MNP (Ecuador)	Galapagos	[43, 44]
Playa Chinchorro	6418ARI	-	-	CmP4.1	Eastern Pacific			
Playa Chinchorro	6518ARI	-	-	CmP5.1	Eastern Pacific			
Playa Chinchorro	6618ARI	-	-	CmP4.7	Eastern Pacific			
Playa Chinchorro	6718ARI	-	-	**CmP4.4**	Eastern Pacific	Galapagos, MNP (Ecuador)	Galapagos	[43, 44]
Playa Chinchorro	6918ARI	-	-	CmP4.6	Eastern Pacific			

Playa Chinchorro	7018ARI	-	-	CmP4.6	Eastern Pacific			
Playa Chinchorro	7118ARI	-	-	CmP4.4	Eastern Pacific			
Playa Chinchorro	7218ARI	-	-	CmP15.1	Eastern Pacific			
Playa Chinchorro	7418ARI	-	-	CmP4.1	Eastern Pacific			
Playa Chinchorro	8118ARI	-	-	CmP4.7	Eastern Pacific			
Playa Chinchorro	8218ARI	-	-	CmP4.7	Eastern Pacific			
Playa Chinchorro	8818ARI	-	-	**CmP4.9**	Eastern Pacific	Galapagos (Ecuador)	Endemic Galapagos	[43, 44]
Playa Chinchorro	8918ARI	-	-	CmP4.6	Eastern Pacific			
Playa Chinchorro	9218ARI	-	-	CmP4.7	Eastern Pacific			
Playa Chinchorro	9418ARI	-	-	CmP4.6	Eastern Pacific			
Playa Chinchorro	1219ARI	-	-	CmP4.4	Eastern Pacific			
Easter Island	902RN	70.8	Juvenile	**CmP4.6**	Eastern Pacific	Galapagos (Ecuador), MNP (Ecuador), Costa Rica, Michoacan (Mexico)	Galapagos	[43, 44]
Easter Island	904RN	80.0	Juvenile	**CmP4.4**	Eastern Pacific	Galapagos, MNP (Ecuador)	Galapagos	[43, 44]
Easter Island	907RN	74.0	Juvenile	**CmP97.1**	Western Pacific	French Polynesia	French Polynesia	[45]
Easter Island	909RN	70.6	Juvenile	CmP97.1	Western Pacific			
Easter Island	911RN	78.6	Male	CmP97.1	Western Pacific			
Easter Island	913RN	55.4	Juvenile	CmP97.1	Western Pacific			
Easter Island	915RN	71.1	Juvenile	**CmP4.1**	Eastern Pacific	Galapagos (Ecuador), MNP (Ecuador), Costa Rica, Michoacan (Mexico), Revillagigedo	Michoacan	[43, 44]
Easter Island	917RN	94.4	Female	CmP97.1	Western Pacific			
Easter Island	919RN	68.5	Juvenile	CmP97.1	Western Pacific			
Easter Island	921RN	58.9	Juvenile	**CmP207.1**	Western Pacific	-	Orphan	[46]
Easter Island	925RN	59.3	Juvenile	**CmP109.1**	Western Pacific	-	Orphan	[46]
Easter Island	927RN	49.0	Juvenile	CmP97.1	Western Pacific			
Easter Island	929RN	64.9	Juvenile	CmP97.1	Western Pacific			
Easter Island	931RN	74.4	Juvenile	CmP97.1	Western Pacific			
Easter Island	933RN	99.0	Female	CmP97.1	Western Pacific			
Easter Island	935RN	95.9	Female	CmP97.1	Western Pacific			
Easter Island	937RN	62.6	Juvenile	**CmP4.14**	Eastern Pacific	-	Orphan	[9]
Easter Island	939RN	64.4	Juvenile	CmP207.1	Western Pacific			
Easter Island	941RN	54.0	Juvenile	CmP207.1	Western Pacific			
